# Supplementary material for: A megawatt ultra-wide bandgap semiconductor module for pulsed power electronics
Source: Nat Commun. 2026 Mar 30;17:4783. doi: 10.1038/s41467-026-71274-6 (PMC13219416; doi:10.1038/s41467-026-71274-6)
Supplement: Supplementary file 1 — Supplementary Information for [file 41467_2026_71274_MOESM1_ESM.pdf]

Supplementary Information

A megawatt ultra-wide bandgap semiconductor module for pulsed power electronics

Hehe Gong[1,#], Xin Yang[1,#], Boyan Wang[2], Zichen Zhang[3], Qingrui Yuchi[3], Zineng Yang[1], Matthew Porter[2], Hongchang Cui[1], Yuan Qin[2], Rong Zhang[4], Han Wang[1], Dong Dong[2], Jiandong Ye[4,*], Guo-Quan Lu[3,*], Yuhao Zhang[1,*]

[1]Centre for Advanced Semiconductors and Integrated Circuits and Department of Electrical and Electronic Engineering, The University of Hong Kong, Hong Kong, SAR, China.
[2]Center for Power Electronics Systems, Virginia Polytechnic Institute and State University, Blacksburg, Virginia 24061, USA

[3]Department of Materials Science and Engineering, Virginia Polytechnic Institute and State University, Blacksburg, Virginia 24061, USA
[4]School of Electronic Science and Engineering, Nanjing University, Nanjing 210023, China

[#]Hehe Gong and Xin Yang contributed equally to this work.
*Corresponding Authors E-mails:
yejd@nju.edu.cn, gqlu@vt.edu, yuhzhang@hku.hk

Supplementary Section S1 - Benchmark of forward current versus breakdown voltage for large-area Ga_2O_3 devices

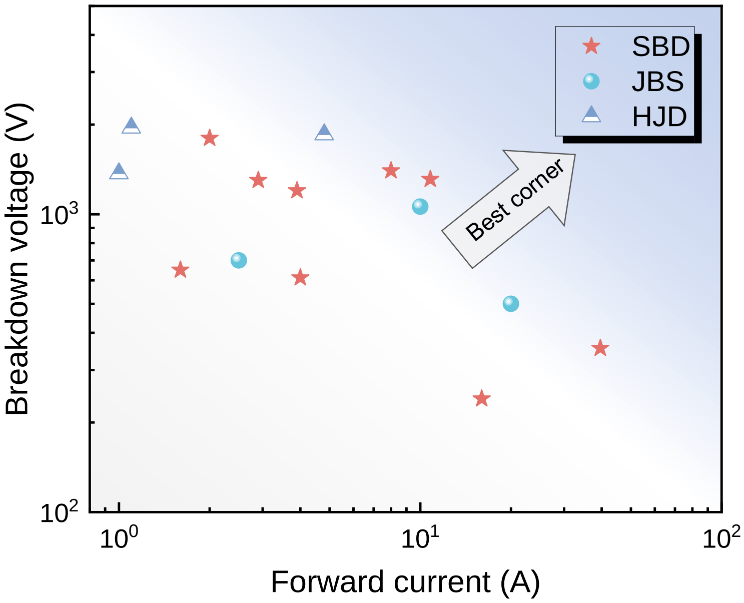

Supplementary Figure S1. Benchmark of forward current (I_F) versus breakdown voltage (V_B) for the reported large-area Ga_2O_3 Schottky barrier diodes (SBDs)[1-8], junction barrier Schottky (JBS) diodes[9-11], and heterojunction diodes (HJDs)[12-14]. For Ga_2O_3 power diodes, the forward current is extracted at a forward voltage of 3 V above the turn-on voltage (V_{ON}). A general trend is observed in which the V_B decreases as the I_F increases in large-area Ga_2O_3 devices. This suggests that the non-uniformity in material properties and fabrication process, as well as the relatively high defect density, are still the dominant performance limiting factors of boosting power in large-area Ga_2O_3 devices.

Supplementary Section S2 – Optical image of overflowed sintered silver

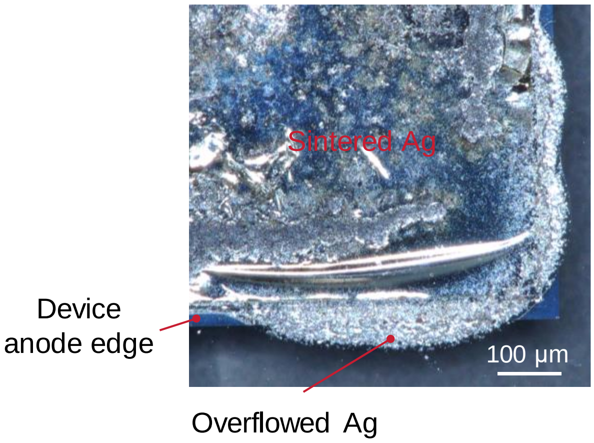

Supplementary Figure S2. Optical image of the decapped contact surface showing sintered Ag on the Ga_2O_3 die. The image was taken after mechanically decapping the co-designed Ga_2O_3 sub-module fabricated following the processes detailed in Supplementary Figure S5. As the silver-sintering paste demonstrated considerable flowability, part of the sintered silver spread beyond the intended anode contact area. This silver overflow resulted in unintended lateral spreading, causing physical contact between the sintered silver and the surrounding dielectric layer. The observed lateral spread length of the silver overflow ranged from approximately 20 to 40 μm. Such overflow might lead to localized electric field crowding or parasitic leakage paths, particularly under high-voltage operation.

Supplementary Section S3 – Extracted *κ* of $BaTiO_3$ film

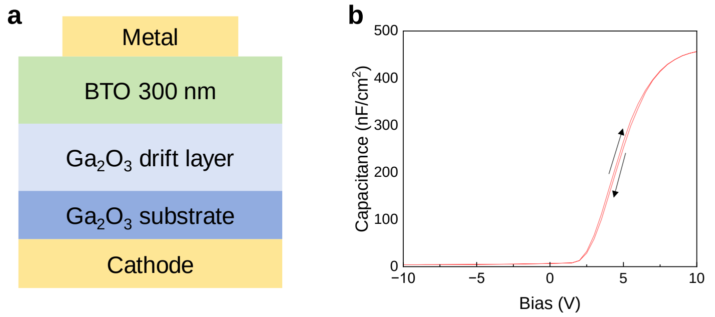

Fig. S3 $BaTiO_3/Ga_2O_3$ MOS-CAP structure and measured C-V hysteresis curves at 100 kHz. The $BaTiO_3$ (300 nm) was deposited by RF magnetron sputtering at room temperature, where the growth pressure was 0.6 Pa in an Ar/O_2 mixed ambient with a flux ratio of 10:1. A 6-µm-thick photoresist was used as the mask, then the anode contact window was opened through a lift-off process. To determine the dielectric constant of $BaTiO_3$, a MOS-CAP with 300-nm-thick $BaTiO_3$ layer was fabricated, and the cross-section schematics is shown in (a). C-V measurements were performed in a DC sweeping voltage range from -10 V to 10 V at 100 kHz, with results shown in (b). At 10 V, the capacitance almost saturates, suggesting the MOS structure enters into the accumulation region. The accumulation capacitance at 10 V is considered as the dielectric capacitance (C_{ox}), and the relative dielectric constant of the $BaTiO_3$ dielectric was calculated as ε_{ox}=155 based on the parallel-plane capacitance equation: $C_{ox} = \varepsilon_{ox}\varepsilon_0/d$, where d is the dielectric thickness.

Supplementary Section S4 - Device fabrication process

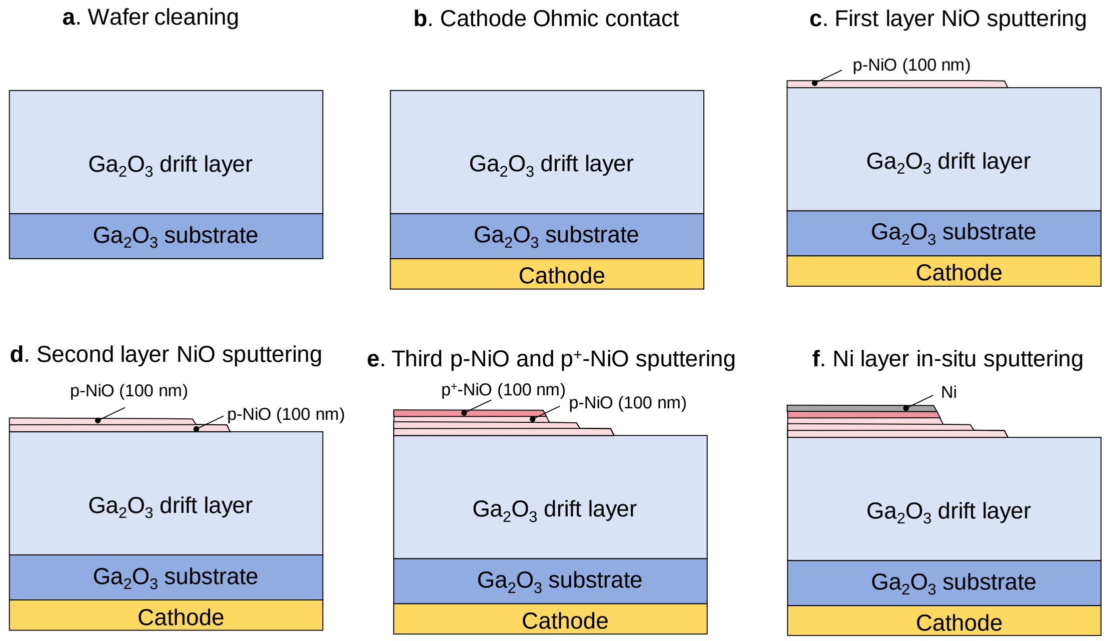

Supplementary Figure S4. Process flow of NiO/Ga_2O_3 heterojunction diode (HJD) fabrication. The main fabrication steps of the NiO/Ga_2O_3 HJDs include (a) wafer cleaning via ultrasonic treatment in acetone and alcohol soaking, followed by annealing Ga_2O_3 epi-wafers at 500 °C under the O_2 ambient. (b) cathode Ohmic metal deposition and followed rapid thermal annealing at 500 °C for 1 min under N_2 ambient, (c) sputtering of first-layered p-NiO (100 nm), the NiO JTE edge presents beveled angle by using a bi-layer photoresist, (d) sputtering of second-layered p-NiO (100 nm), (e) third-layered p-NiO (100 nm) and p^+-NiO (100 nm), (d) in-situ sputtering of metal Ni layer (100 nm).

Supplementary Section S5 – Package process

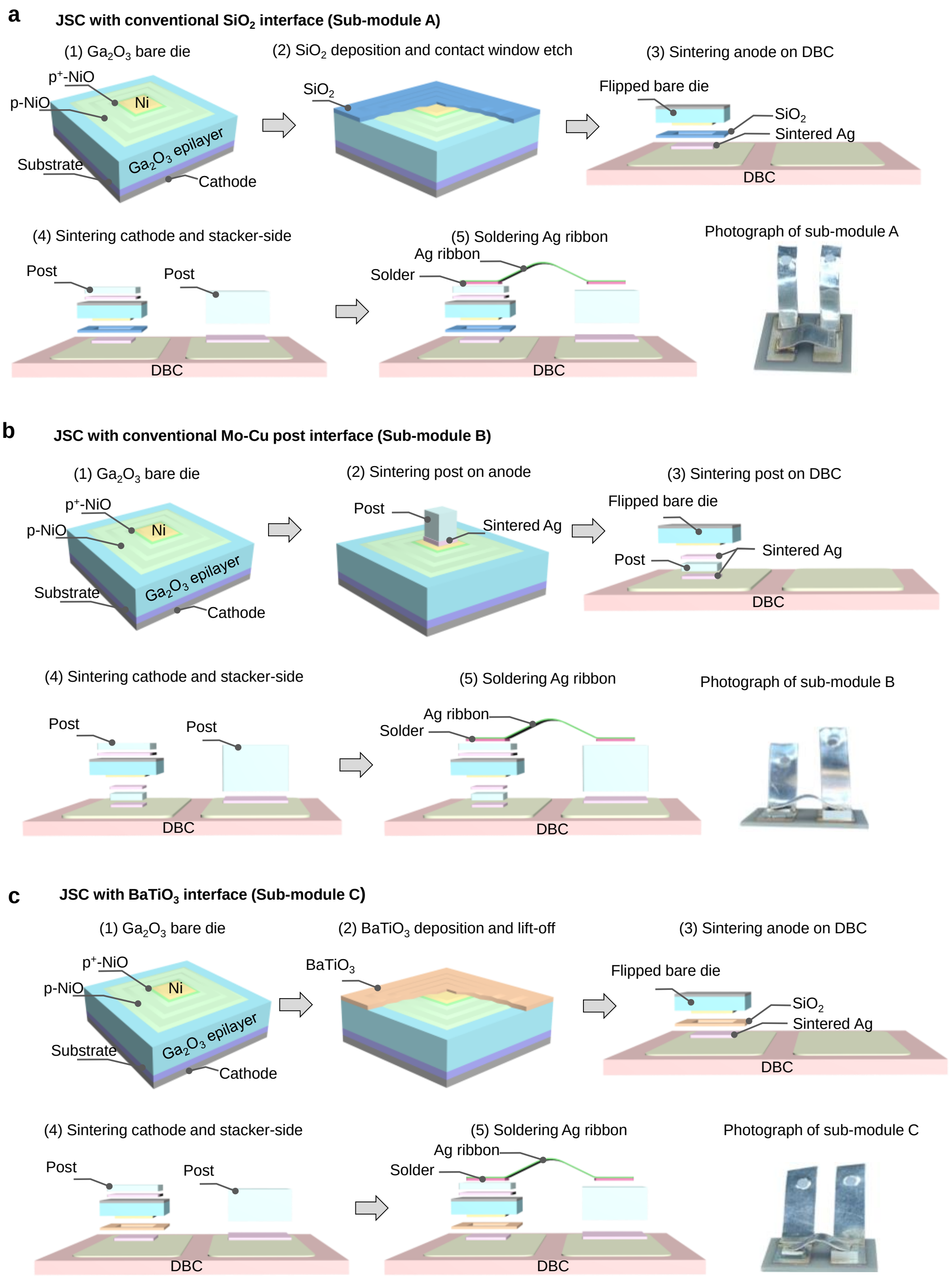

Supplementary Figure S5. Process flows JSC packaging of three sub-modules (A, B and C). The package methodology was designed to comparatively evaluate the effects of different JSC (junction-side cooling) interface schemes on the electrical and thermal performance of submodules.

DBC Patterning

Prior to die attachment, the AlN-based direct bond copper (DBC) substrate (provided by Rogers Corporation) was patterned to define the electrode layout. The patterning process involved applying Kapton tape as a hard mask, selectively removing unwanted regions using precision laser cutting, and then performing wet chemical etching with sprayed $FeCl_3$ solution. This method ensured clean, well-defined metallization features on the DBC with minimal undercutting and damage to the ceramic base.

Sub-module Definitions

All three sub-modules are with JSC package. Sub-module A features a conventional SiO_2 interface

layer to isolate semiconductor dies with die attach materials. Sub-module B features a Mo-Cu composite post to physically distance the Ga_2O_3 die from the DBC substrate, aiming to avoid overflow of sintered Ag and enhance blocking capacity. Sub-module C utilizes a $BaTiO_3$ interface, intended to suppress field crowding at the edge, as well as improve the thermal performance compared to Sub-module B.

Package process in Sub-module A and C

For Sub-modules A and C, a dielectric engineering approach is applied at the Ga_2O_3 anode surface. Specifically, a 300 nm SiO_2 layer (Sub-module A) and a 300 nm $BaTiO_3$ layer (Sub-module C) are deposited by plasma-enhanced chemical vapor deposition (PECVD) and RF magnetron sputtering, respectively. The $BaTiO_3$ was deposited by RF magnetron sputtering at room temperature, where the growth pressure was 0.6 Pa in an Ar/O_2 mixed ambient with a flux ratio of 10:1. The RF power used was 150 W, and the target was high purity (99.99%) $BaTiO_3$ ceramics.

After dielectric deposition, a contact window in $BaTiO_3$ is opened via a photolithography and lift-off process. A contact window in SiO_2 is opened by photolithography and dry-etch process. The device top and bottom electrode metal finish are formed by evaporating an Au/Ti (200 nm) bilayer. The Ti layer serves as a diffusion barrier to prevent silver migration into the Ga_2O_3 during subsequent sintering. Die attach was carried out by stencil-printing a ~50 μm-thick silver-sintering paste (NBE Technologies) onto the anode using a laser-cut polyimide mask. Then the die is placed on the patterned DBC and pressurelessly sintered in the air. The sintering temperature ramps from room temperature to 230 °C at 6 °C /min, holds at 230 °C for 1 hour, and is then cooled to room temperature in air. This sintering condition enables densification and metallurgical bonding without the need for external pressure, preserving interface integrity.

Package process in Sub-module B

For Sub-module B, a Mo-Cu composite post ($2.5 \times 2.5\ mm^2$ contact area, 0.5 mm thick) was first silver-plated to enhance bonding. It was sintered onto the Ga_2O_3 anode using the same silver-sintering paste and pressureless sintering profile as above.

The subsequent steps, e.g., cathode and stacker-side sintering, soldering and ribbon formation, are the same for all sub-modules. A Mo-Cu post ($4 \times 4 \times 0.5\ mm^3$) is sintered onto the cathode side of the Ga_2O_3 die, also using silver sintering. The stacker-side post assemblies with height similar to the Ga_2O_3 chip thickness are simultaneously sintered onto the pre-patterned DBC. Silver ribbons are soldered to the top surfaces of both Mo-Cu posts using Sn-42Pb-8Bi solder paste to serve as the main electrical interconnects. Additional silver ribbons are soldered to the DBC pads to act as external leads.

Encapsulation

The last step in the module assembly process was encapsulation for the thermal resistance measurement and circuit test. A 3D-printed engineering-grade nylon housing was glued to the DBC surface using a slow-curing epoxy resin (Bob Smith Industries). The housing mainly serves as a mechanical support and fixture to form a cavity for gel filling. A two-part silicone gel (Wacker 612 A/B) was mixed, vacuum-degassed to eliminate air bubbles, and poured into the housing to fully encapsulate the device and interconnects. The silicone gel is used to provide isolation and anti-vibration protection and prevent moisture or ionic contamination. The assembled modules were then cured at 150 °C for 30 minutes on a hotplate to complete the packaging process. The junction temperature during transient power may momentarily reach 250-300 °C. But the external package temperature remains much lower because of the extremely short pulse width and the low thermal diffusivity between junction and housing. Therefore, the over 200 °C operation capability demonstrated in Fig. 2 reflects an intrinsic property of the Ga_2O_3 device itself and the JSC stack, not of the external housing or silicone gel.

Supplementary Section S6 – Physics-based TCAD simulation

The physics-based Technology Computer Aided Design (TCAD) simulation is performed using Silvaco. The goal is to analyze electric field distribution across the entire internal package under reverse bias conditions (anode bias at -2 kV). Table S1 lists the basic material parameters used in the simulation for the Ga_2O_3 submodule with different configurations. The used simulation models include the Chynoweth impact ionization model, field-dependent mobility, Shockley-Read-Hall recombination, Auger recombination, and bandgap narrowing effects. Poisson's equation and carrier transport equations were solved self-consistently under steady-state and transient bias conditions. Mesh refinement was applied in high-field regions and various material interface to ensure numerical accuracy. A non-uniform mesh with high resolution near the hetero-interface was employed to accurately capture electric field gradients, mesh refinement was also applied in high-field regions.

Table S1. Basic material parameters used in the simulations.

Material	Key parameters
Ga_2O_3	Permittivity (ε_{GaO}):12.4 (plane (001))<br>Band-gap: 4.8 eV<br>Affinity: 4.1 eV<br>Donor concentration: 1.5×10^{16} cm^{-3} (Drift layer), 5×10^{18} cm^{-3} (Substrate)
NiO	Permittivity (ε_{NiO}): 11.9<br>Band-gap: 3.8 eV<br>Affinity: 2.0 eV<br>Acceptor concentration: 1.7×10^{17} cm^{-3} (p-NiO), 2×10^{19} cm^{-3} (p^+-NiO)
$BaTiO_3$	Permittivity (ε_{BTO}): 155<br>Band-gap: 3.4 eV
SiO_2	Permittivity (ε_{SiO2}): 3.9<br>Band-gap: 9 eV

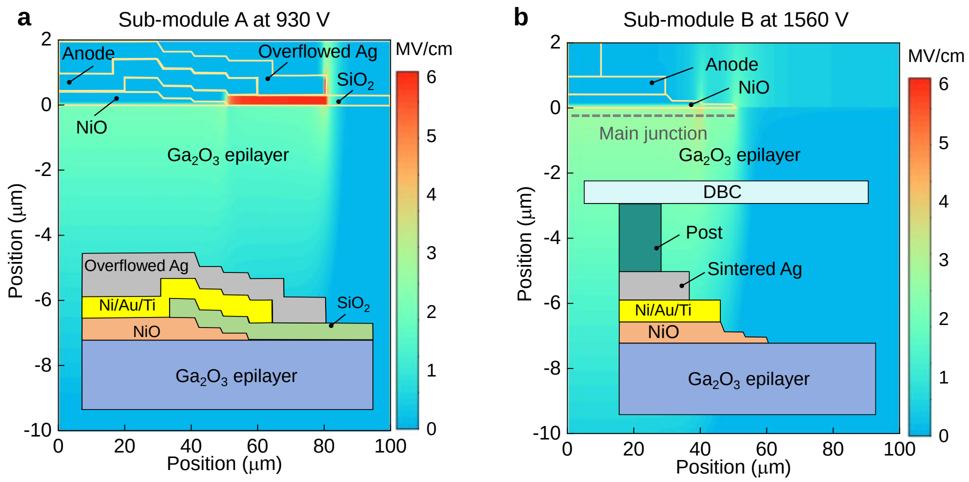

Supplementary Figure S6. Electric field simulation. Two-dimensional electric field contours in JSC-packaged NiO/Ga_2O_3 heterojunction devices (a) with a SiO_2 interface (control sub-module A) at 930 V, (b) with a post interface (control sub-module B) at 1560 V.

To further verify consistency between the simulated and measured breakdown voltages, we have

added electric-field contours simulated at the actual breakdown points. As shown in Fig. S6, at 930 V for sub-module A, the peak electric field within the SiO_2 layer reaches approximately 11.7 MV/cm, which exceeds the dielectric strength of SiO_2 and confirms that breakdown is governed by dielectric failure. For sub-module B, at its measured breakdown voltage of 1560 V, the peak electric field at the main junction region is approximately 4.2 MV/cm, which is significantly higher than the average junction field. Given the large device area (3 mm × 3 mm), localized leakage paths can readily initiate in this high-field region, ultimately leading to breakdown.

Supplementary Section S7 - Temperature-dependent breakdown characterization of SiC SBD

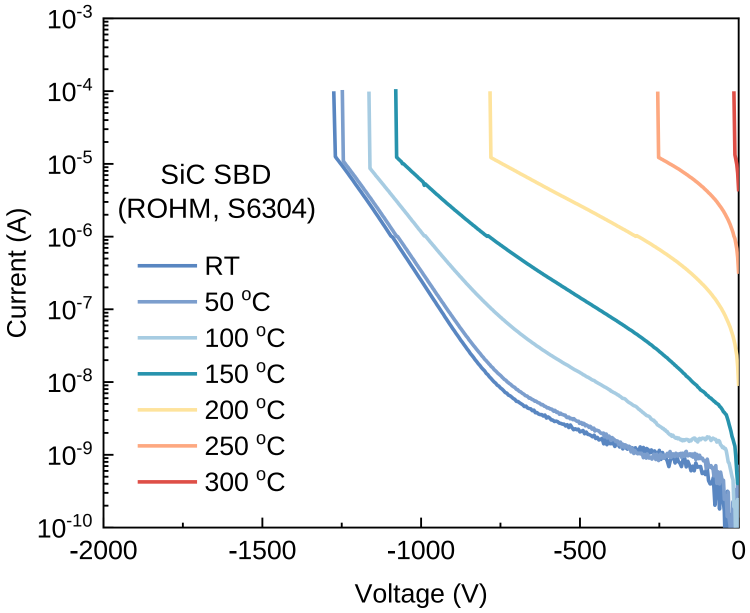

Supplementary Figure S7. Temperature-dependent reverse I-V characteristics of a bare-die SiC Schottky barrier diode (SBD) from room temperature to 300 °C. The data correspond to a commercial 1200-V rated SiC SBD (ROHM, S6304). The SiC SBD exhibits higher reverse leakage current than the Ga_2O_3 HJD, especially as the temperature increases over 200 °C. The SiC reference device used in Supplementary Section S7 is a commercial 1.2-kV SiC junction barrier Schottky (JBS) diode with a drift layer thickness comparable to our Ga_2O_3 device. Note that it is used to represent a typical industrial SiC SBD in the close voltage class, rather than the ultimate capability of high-voltage SiC bipolar devices that has been reported to be able to operate at very high temperatures[15]. The device combines Schottky regions with buried p^+ grids to suppress leakage. Under reverse bias, especially at high temperature, leakage is still predominantly governed by the metal/SiC Schottky barrier, leading to a pronounced increase of leakage with temperature.

Supplementary Section S8 – Thermal impedance measurement

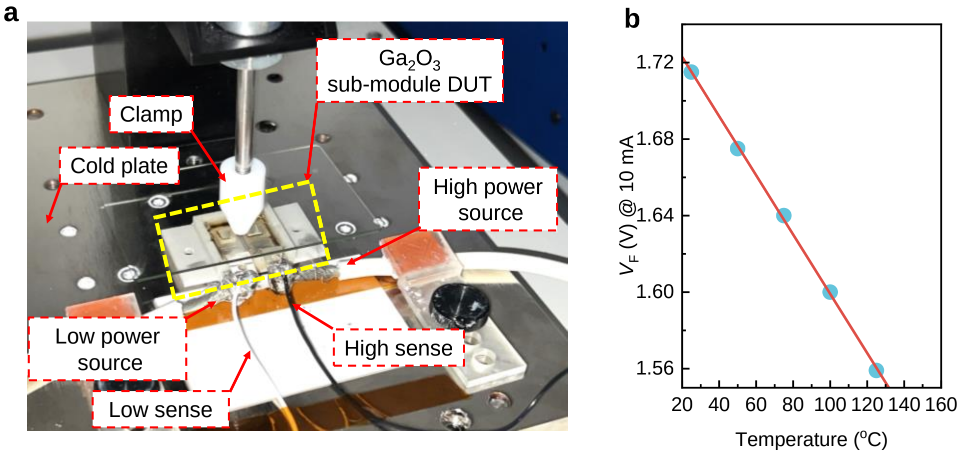

Supplementary Figure S8. Thermal resistance measurement setup. The junction-to-case thermal impedance ($Z_{\theta JC}$) is investigated using the transient dual interface method (TDIM) based on the JEDEC 51-14 standard[16]. This TDIM method relies on two transient thermal impedance curves (Z~t) measured with different contact thermal resistances between the package case surface and the ambient. The Z value at the separation point of the two curves is close to the device steady-state junction-to-case thermal resistance ($R_{\theta JC}$). This method avoids the errors caused by traditional thermocouple methods, and has been widely used for power devices and modules.

In the TDIM method, the junction temperature (T_j) is usually monitored by continuously measuring a thermo-sensitive electrical parameter (TSEP)[17]. TSEP was extracted from the diode's current-voltage curve, which correlates the forward voltage drop (at forward current of 10 mA) with junction temperature during transient thermal measurements. This forward voltage drop has been found to exhibit a linear dependence on temperature from the calibration measurements performed in a thermal chamber (Fig. S8-b). This linearity confirms its validity as an accurate TSEP.

During the measurement, the Ga_2O_3 sub-module was placed on a water-cooling cold plate with a fixed temperature. A top plastic clamp applied a ~15-psi pressure to ensure good and consistent contacts. As this clamp has very low k_T, this setup allows heat extraction dominantly towards the bottom water-cooling plate. The $Z_{\theta JC}$ measurements started by applying a large forward dc bias to the device for self-heating, until the steady state was reached with a constant T_j (T_{j0}). Subsequently, the dc power was cut off, and the TSEP was monitored to obtain the evolution of $T_j(t)$ in the cooling phase. The Z~t curve was calculated by $Z_{\theta JC}(t)=(T_{j0}-T_j(t))/P_H$, where P_H is the heating power. For each sub-module $Z_{\theta JC}$ test, two Z~t curves were acquired by using two different thermal interface materials between the module case and the cold plate. In this work, we used silicone oil as a lower k_T interface and thermal grease as a higher k_T interface. Data analysis following the JEDEC standard will allow the extraction of transient thermal impedance and the steady-state thermal resistance, with the latter determined from the separation point of the two structure function curves.

Supplementary Section S9 - ANSYS Workbench simulation and calibration with experimental circuit test results

To investigate the internal thermal distribution and the maximum junction temperature in the Ga_2O_3 packaged device, we simulate multiple Computational Fluid Dynamics (CFD) cases using ANSYS Icepak based on ANSYS Workbench, which uses a Finite Volume Method (FVM) for numerical solutions.

(1) Basic material parameters

Table S2 lists the basic material parameters used in the thermal simulation for the Ga_2O_3 module with different configurations.

Table S2. Basic material parameters used in thermal simulations.

Material	Specific heat capacity (J/g K)	Thermal conductivity (W/m K)	Density (g/cm^3)
Ga_2O_3	0.56	20	5.96
Sintered Ag	0.24	180	7.5
Post (Mo-Cu)	0.3	150	9.5
Silicone gel.	1.5	0.25	0.98
AlN DBC	0.74	160	3.26

(2) Calibration with circuit experiments

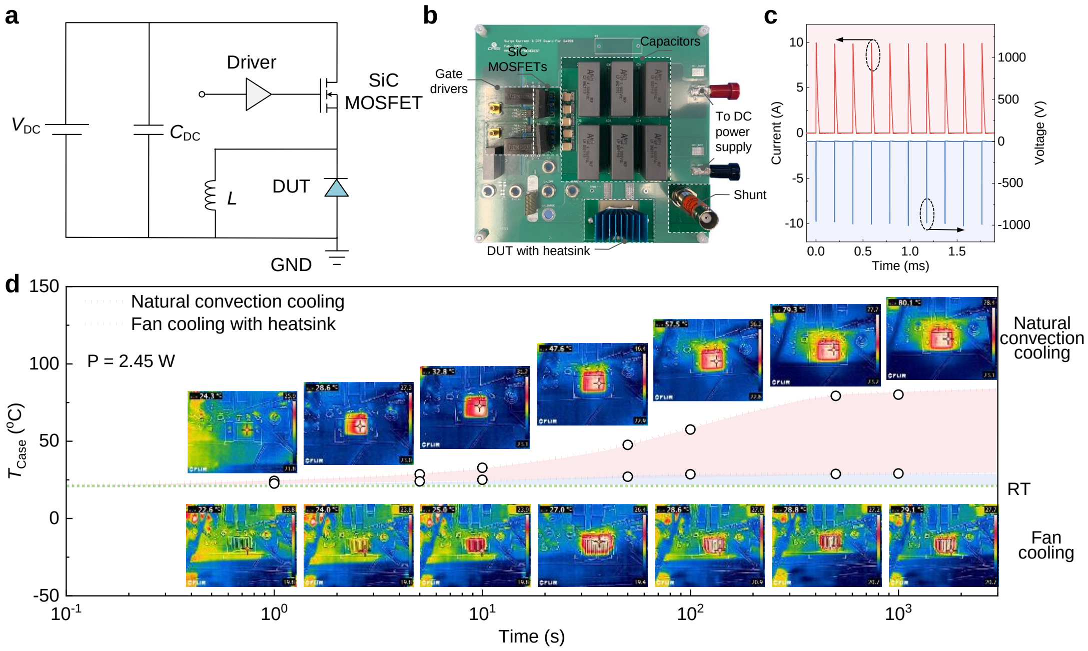

Supplementary Figure S9. (a) Schematic and (b) photo of the dynamic switching circuit test setup. (c) Switching current waveforms and voltage waveforms. (d) Recorded case temperature during the circuit operation from 1s to 1000 s under natural convection cooling and fan cooling with heatsink, as well as the simulated junction temperature evolution shown in dashed lines for two cooling schemes.

The thermal simulation models were calibrated using experimentally recorded case temperatures obtained from a thermal camera under continuous device switching in circuit. The circuit consists of a dc bus voltage, a large bus capacitor, an active switch (1.2 kV-rated SiC MOSFET), an inductive load, and the device under test (DUT), *i.e.*, Ga_2O_3 sub-modules. The SiC MOSFET is controlled by a gate driver. The DUT undergoes continuous switching between 10 A and 1000 V, with a switching frequency of 5 kHz. Two circuit tests are performed under different cooling schemes, one under natural convection

cooling and the other under fan cooling with heatsink. The switching circuit is modulated to deliver a constant power of 2.45 W dissipated in the diode module, and the resulting surface temperature was recorded from 1 s to 1000 s after the initiation of circuit operation.

As shown in Supplementary Fig. S9d, the simulated case temperatures (dashed lines) closely matched the measured values over this time range, spanning three orders of magnitude, under both cooling schemes. This excellent agreement between simulation and experiment validates the thermal model parameters, including thermal boundary conditions and material properties. Based on this calibration, we extend confidence in the simulation results for shorter time scales (1 μs to 1 s), where direct thermal camera measurements are not feasible. This calibrated thermal simulation enables reliable estimation of the rapid, internal thermal evolution during dynamic switching events, which is critical for understanding electro-thermal stress in Ga_2O_3 power modules.

Supplementary Section S10 – Power capacity modeling

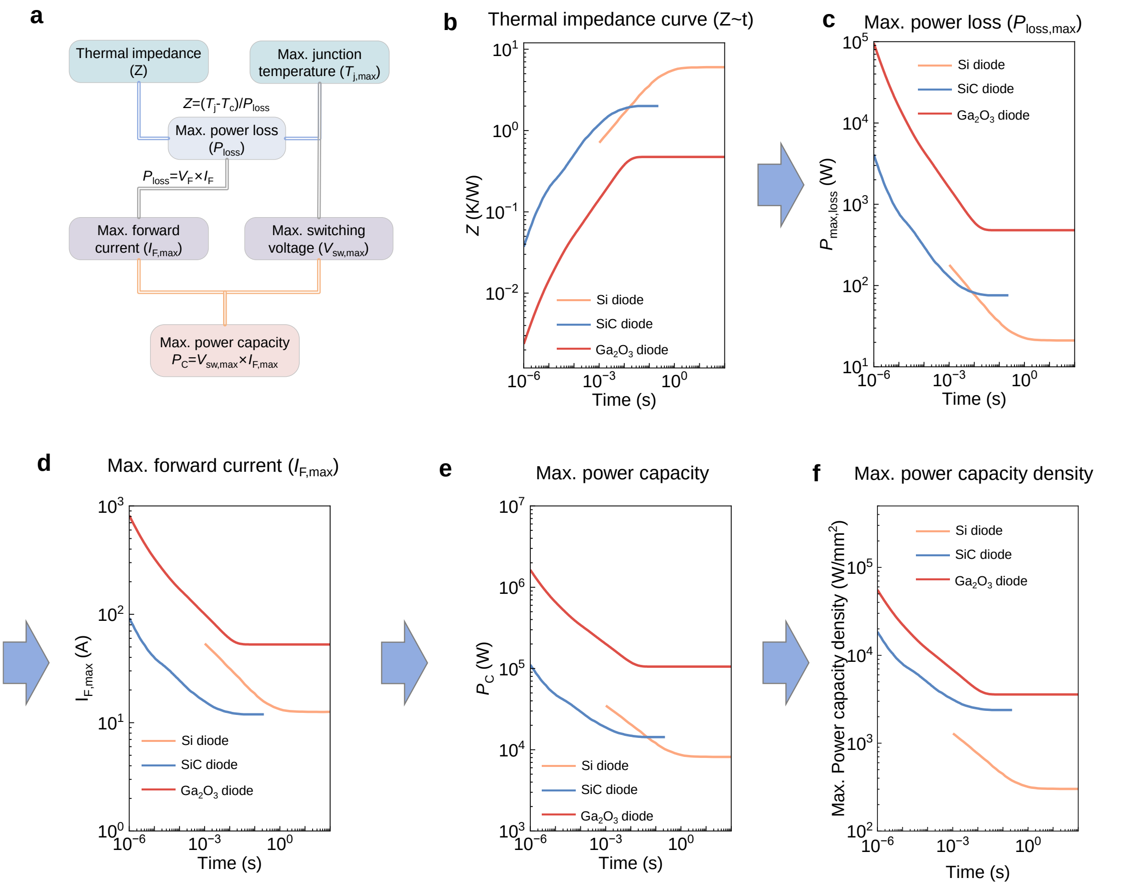

Supplementary Figure S10-1. Diagram of power capacity modeling. (a) Diagram of the developed model to compare the maximum power capacity of Ga_2O_3, SiC, and Si diodes under pulsed operation, based on experimental data including transient thermal impedance (Z~t) curves, maximum T_j, and I-V-T characteristics. Here we compare the maximum P_C of Ga_2O_3 diode sub-module with two state-of-the-art industrial, packaged SiC and Si diodes with the same differential on-resistance (which indicates a similar current rating). The part numbers of SiC and Si diodes are IDH02G120C5 and RF305BM6S, respectively. (b)-(f) Detailed step-by-step derivations as illustrated below.

Step1: Extraction of short-pulse transient thermal behavior (Fig. S10(b))

Under short-pulse conditions, the heat generated in the semiconductor die does not fully reach the case or heatsink. Therefore, the case temperature (T_C) can be approximated as constant and equal to the ambient temperature. This allows the estimation of transient junction temperature $T_j(t)$ rise based on the device's instantaneous power dissipation $P_{loss}(t)$ and transient thermal impedance $Z(t)$ using the relation: $Z(t)=(T_j(t)-T_C)/P_{loss}(t)$. For Ga_2O_3 sub-module, the transient thermal impedance is extracted from Z~t curves in Fig. 3a. For SiC and Si diodes, the transient Z~t is extracted from their datasheets.

Step 2: Determination of maximum allowable power dissipation (Fig. S10(c))

For each device type, the maximum allowable junction temperature $T_{j,max}$ is used to determine the corresponding maximum instantaneous power dissipation before degradation or failure.

Ga_2O_3 diode: $T_{j,max}$ = 250 °C (based on Fig. 2d)

SiC diode: $T_{j,max}$ = 175 °C (based on datasheet and application note)

Si diode: $T_{j,max}$ = 150 °C (based on datasheet and application note)

Given the transient thermal impedance Z(t), the time evolution of maximum power loss is calculated by

$P_{loss,max}(t) = (T_{j,max}-T_C)/Z(t)$.

Step 3: Extraction of maximum forward current (Fig. S10(d))

The maximum forward current ($I_{F,max}$) can be obtained from the device *I-V* curves in the linear conduction region, $P_{loss,max}(t)=V_{F,max}(t)*I_{F,max}(t)$. Here the *I-V* curve is extracted at the respective $T_{j,max}$ for each diode. For Ga_2O_3 sub-module, the *I-V* curves are shown in Fig. 2c. For SiC and Si diodes, their *I-V* curves are obtained from the respective datasheet.

Step 4: Derivation of maximum power capacity (Fig. S10(e))

The maximum power capacity $P_{C,max}$ of each device under pulse operation is calculated by $P_C = V_{SW,max}*I_{F,max}$, Where $V_{SW,max}$ is the maximum switching voltage (usually the rated voltage) under the maximum junction temperature. The maximum switching voltages considered here are:

Ga_2O_3: 1700 V (at $T_{j,max}$ = 250 °C) (based on Fig. 2d, ~85% of the avalanche breakdown voltage, accounting for a safe overvoltage margin in practical applications)

SiC: 1200 V (at $T_{j,max}$ = 175 °C) (based on datasheet and application note)

Si: 650 V (at $T_{j,max}$ = 150 °C) (based on datasheet and application note)

Step 5: Derivation of maximum power capacity density (Fig. S10(f))

For a fair comparison, the Ga_2O_3 diode sub-module is benchmarked against SiC and Si diodes for the maximum power capacity density, defined as $P_{C,max}$ normalized by the device's active area. This metric allows for a direct evaluation of the intrinsic power-handling capability per unit area across different device platforms.

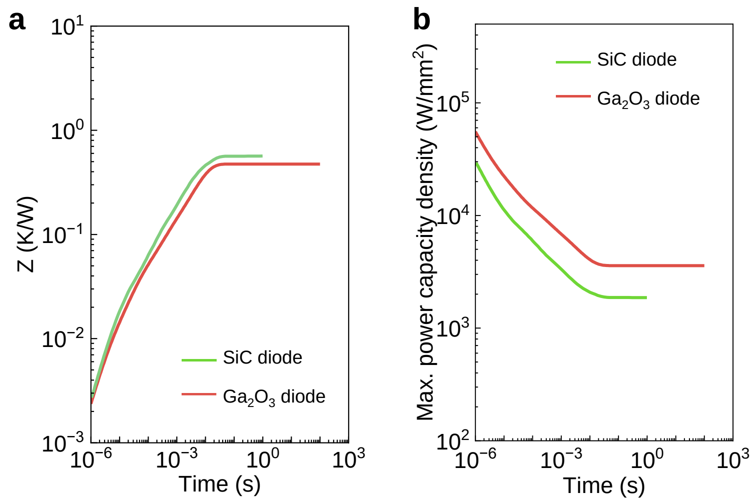

Supplementary Figure S10-2. Thermal impedance and maximum power capacity density. (a) Derived transient thermal impedance of the Ga_2O_3 sub-module and packaged SiC diode (IDWD20G120C5) across various time scales. (b) Extracted maximum transient power capacity density as across varying time scales.

Finally, we discuss the impact of packages and device selections on the above comparative analysis. First, from the later discussion in Supplementary Section S14, in pulsed power applications, the shorter the pulse width, the device maximum power capacity density is expected to be less dependent on packages but dominantly determined by material's inherent heat capacity and device's maximum operation temperatures. Thus, the TO package of SiC diode and the JSC package of our Ga_2O_3 diode will present relatively minimal difference for the thermal transients at short pulse duration. Second, to investigate the analysis' dependence on device selection, we select a higher-current rated commercial

SiC diode (IDWD20G120C5, Infineon) for a new comparison, which exhibits a high continuous forward current of I_F= 61 A and a non-repetitive surge forward current of I_{FSM}= 190 A. This thermal resistance is nearly identical to that of our Ga_2O_3 sub-module, enabling a fair comparison. As shown in Figure S10-2(a) and (b), the Ga_2O_3 sub-module still demonstrates significantly higher maximum transient power capacity density across the entire time window. This suggests, even if SiC diodes are scaled to larger die areas, their transient maximum power capacity density remains fundamentally limited by SiC material properties.

Supplementary Section S11 – Experimental circuit testing setup for short-pulse DPT

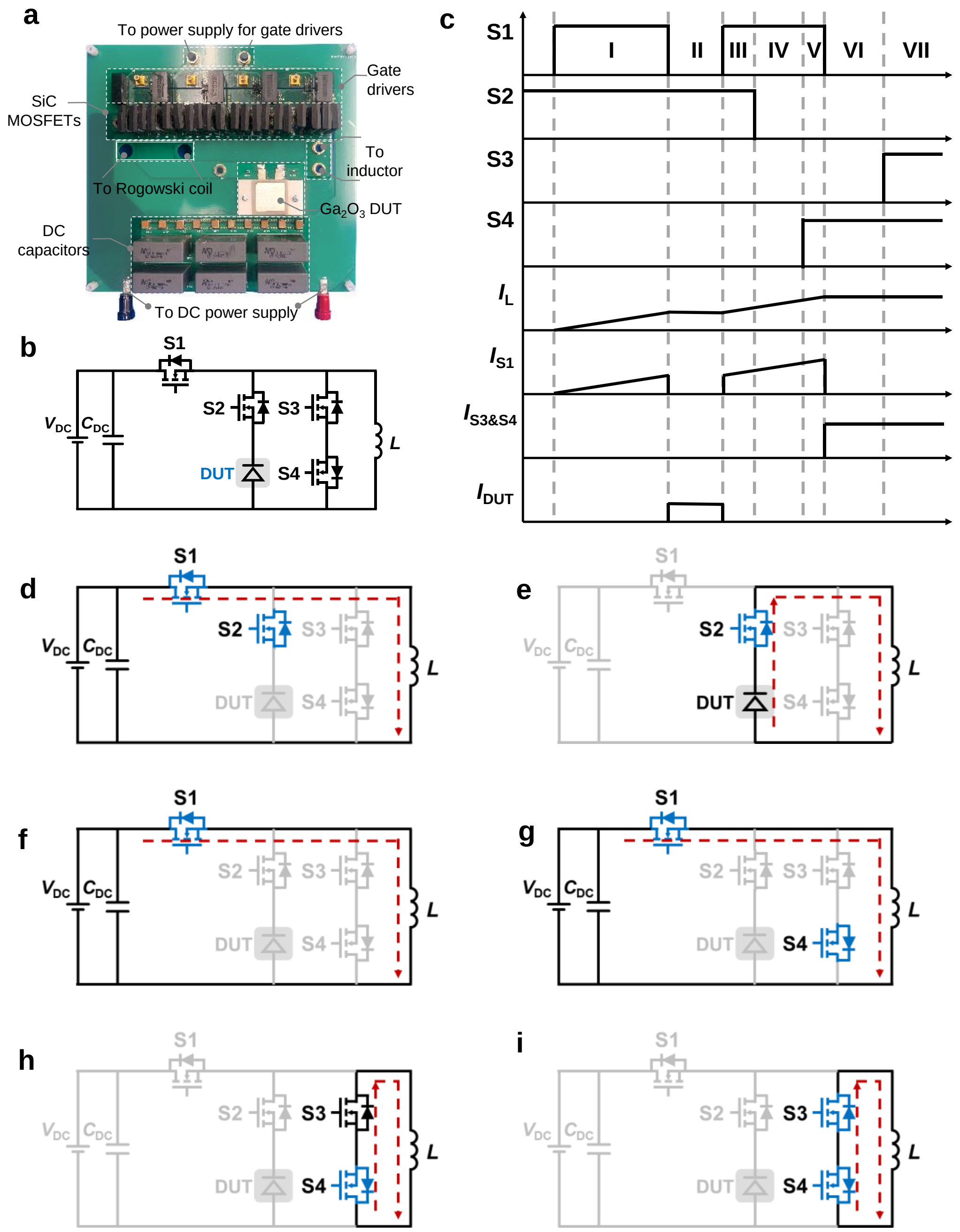

Supplementary Figure S11. Short pulse DPT circuit and waveforms. To produce a high current pulse followed by a high voltage bias for the diode under test (DUT), a custom-designed double-pulse test circuit has been developed. (a) photograph and (b) circuit schematic of the proposed test setup, which includes a DC power supply (V_{DC}), DC capacitance (C_{DC}), a load inductor (L), four power switches (S1-S4), and the Ga_2O_3 DUT. The part number of the SiC MOSFETs is C3M0016120D from Wolfspeed, the rated voltage is 1200 V and the typical on-resistance (R_{on}) is 16 mΩ. The DUT is connected in series with power switch S2, while switches S3 and S4 are also connected in series. These two branches are paralleled to the load inductor, providing two potential free-wheeling paths based on the control signals of the power switches. (c) the ideal control and switching waveforms, detailing the control logic for the four switches, inductor current (I_L), switch currents ($I_{S1\text{-}S4}$), and the device current (I_{DUT}).

The operation of the circuit is divided into seven stages. In Stage I (**Figure S11d**), switches S1 and S2 are turned ON, allowing V_{DC} to charge the inductor through S1, which generates a linearly increasing current in both S1 and L. The ON state of S2 offers a free-wheeling path, rendering the DUT to stay in a reverse-biased condition in this stage. In Stage II (**Figure S11e**), S1 turns OFF, I_L switches to the free-wheeling path formed by the DUT and S2. Due to the lower on-state voltage of S2 and the DUT compared to V_{DC}, the current remains nearly constant, effectively creating an ideal pulse current for the DUT. When S1 turns ON again (**Figure S11d**), the states of the four switches are the same as Stage I. S2 remains ON to provide redundancy during the device's switching transients and to ensure control safety. With a delay of 100 ns, S2 turns OFF, transitioning into Stage IV as shown in **Figure S11f**. A dead time is set to turn ON S4 after S2 turns OFF. In Stage V (**Figure S11g**), S4 turns ON in advance to establish a free-wheeling path for I_L, replacing the original path comprised of the DUT and S2. When S1 turns OFF in Stage VI (**Figure S11h**), I_L flows through S4 and S3. Although S3 is OFF during this stage, its body diode conducts the current. In Stage VII (**Figure S11i**), S3 turns ON to reduce conduction loss, and I_L decreases steadily until it reaches zero.

Supplementary Section S12 – Thermal images in circuit operation

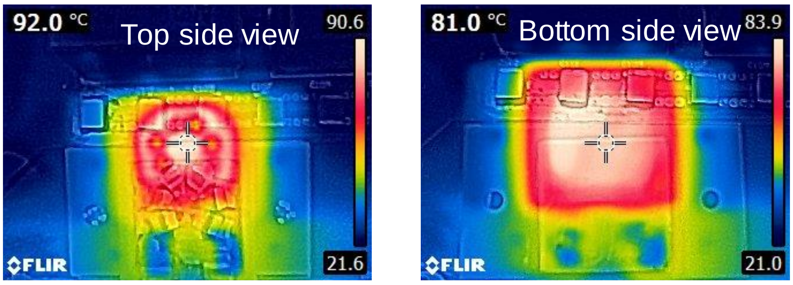

Supplementary Figure S12. Thermal images of (a) top and (b) bottom Ga_2O_3 full module surfaces. A continuous switching stress test was performed under 1,000 V/400 A operating condition with forced-air cooling. The case temperature reached 92 ºC on the top and 81 ºC on the bottom at steady state. From the top-side view, the uniform thermal distribution verifies the uniform current sharing.

Supplementary Section S13 – Experimental circuit setup for surge current test

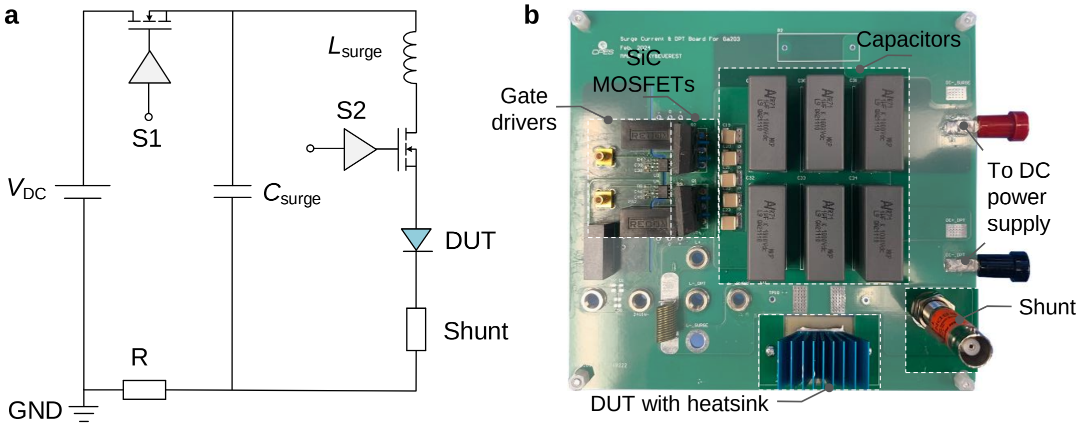

Supplementary Figure S13. Experimental circuit setups. (a) Circuit schematic and (b) photo of a surge current test circuit. The surge test circuit and dynamic switching circuit share the same PCB layout as shown in Fig. S9-2b. By modifying the connected inductance, capacitance, and wiring configuration, different functionalities can be realized on the same hardware platform. In the circuit system, the surge test circuit consists of two primary functional loops:

Power loop: This loop includes the DC power supply (V_{DC}) and the pulse-forming inductor (L_{surge}) and capacitor (C_{surge}). This loop stores and delivers energy during the pulse event.

Driver loop: The Si8271 isolated gate drivers are used to control the gate of the 1200 V-rated SiC MOSFETs (Wolfspeed, C3M0075120D), which act as the high-speed switching devices in the surge circuit.

The diode under test (DUT) is connected in the main current path. The DUT's voltage is measured using a Tektronix THDP0200 high-voltage differential probe, while the current is monitored via a low-inductance 0.01 Ω shunt resistor.

Pulse width control method

The pulse width in the surge circuit is primarily determined by the resonance behavior of the LC components and the switching control of the SiC MOSFET. The basic principle can be understood as follows:

Stage I (Energy Storage): When the first SiC MOSFET (S1) is turned on, the C_{surge} is charged to the input voltage (V_{DC}), storing energy in the form of electrostatic potential.

Stage II (Pulse Initiation): When the second SiC MOSFET (S2) is turned on, the energy stored in C_{surge} begins to discharge through the inductor L_{surge} and the DUT, creating a rising current and a surge-like voltage profile across the device.

Stage III (Pulse Shaping): The inductor L_{surge} determines the rate of current rise (di/dt), while the capacitor C_{surge} affects how quickly the voltage collapses. The interaction of these two components defines the natural LC response time: $t_{pulse}=\sqrt{L_{surge}\cdot C_{surge}}$. By varying L_{surge} and C_{surge}, the characteristic time constant of the surge pulse can be controlled, thus enabling adjustable pulse widths.

Stage IV (MOSFET Turn-Off): The SiC MOSFET is turned off at a predefined delay or voltage threshold, further shaping the pulse duration if required. In underdamped LC conditions, a single current peak is typically observed.

This passive-control approach offers a simple and effective way to tune the surge pulse width without relying on active waveform generators or complex timing circuits. It is particularly useful for

evaluating the transient thermal and electrical robustness of power devices under fast-rising, high-energy pulses. In our setup, different inductors and capacitors can be manually swapped or switched via jumpers or relays, allowing pulse width tuning across a wide range (3.6 μs to 10 ms). The surge current and peak power can also be controlled simultaneously by adjusting V_{DC}.

Supplementary Section S14 - Simulated temperature contours in surge current stress

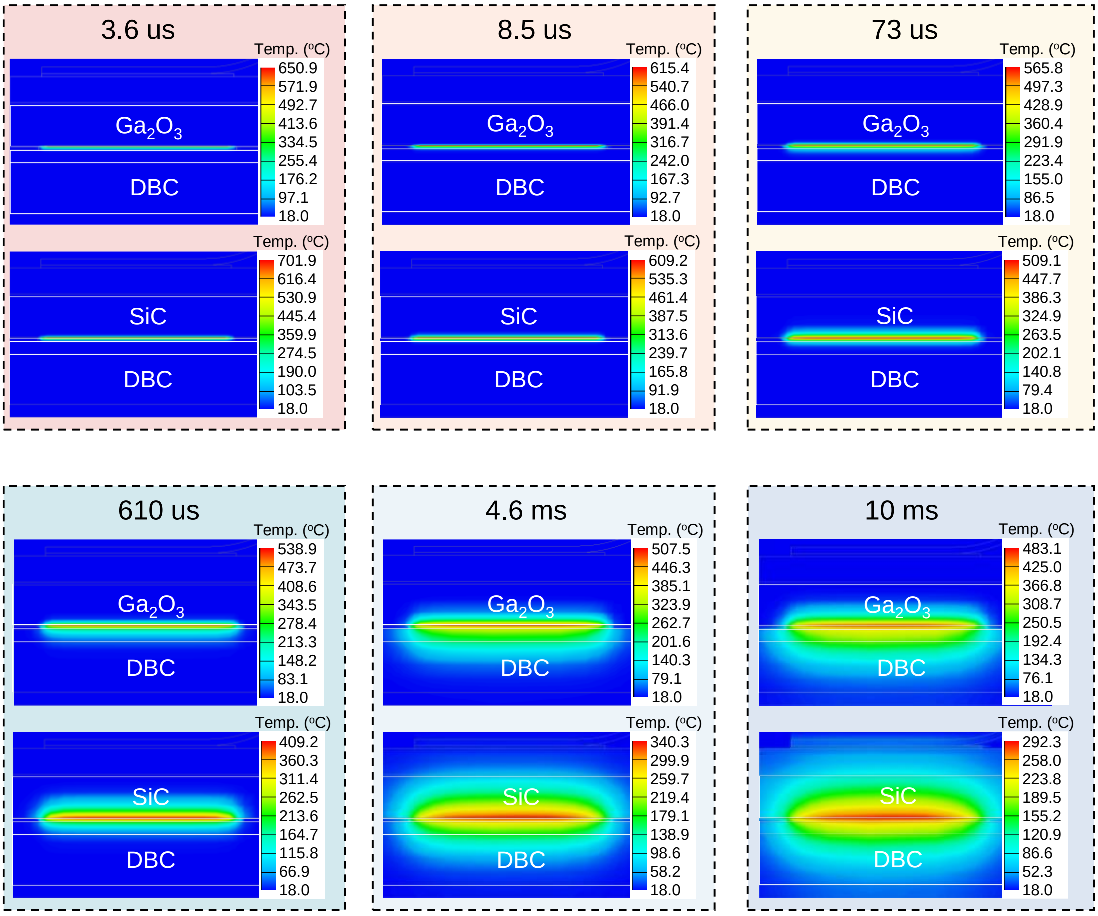

Supplementary Figure S14. Simulated temperature distribution in the Ga_2O_3 sub-module and the hypothetical SiC sub-module in the surge current test under different pulse widths; the peak temperature scenario (i.e., under critical surge current test below the failure boundary) is shown for each pulse width.

To gain deeper physical insight into the role of material properties in transient thermal behavior, we conducted three-dimensional transient thermal simulations in ANSYS for two sub-modules with identical device and packaging structures. One sub-module is the optimized Ga_2O_3 sub-module, while the other is a hypothetical sub-module that keeps all structures but changes the material properties to SiC. Both sub-modules were subjected to the same experimentally extracted surge power pulses, and the internal temperature evolution was tracked over varying pulse widths.

Supplementary Figure S14 shows the simulated temperature contour maps for surge current tests with different pulse widths. At long pulse widths (e.g., 10 ms), the SiC sub-module exhibits lower peak junction temperatures compared to the Ga_2O_3 sub-module, primarily attributed to SiC's higher thermal conductivity. In contrast, at short pulse widths (e.g., 3.6 μs), the Ga_2O_3 sub-module demonstrates a lower junction temperature than the SiC counterpart. This counterintuitive behavior originates from Ga_2O_3's higher volumetric heat capacity (C_v), which enables superior energy absorption in short thermal transients. These results underscore the dynamic tradeoff between thermal conductivity and heat capacity in pulsed power operation and highlight the critical importance of a material's C_v – and the total thermal capacitance of the system — in governing short-duration thermal performance. The temperature contour evolution shown here directly supports the critical T_j trends summarized in Fig. 5h.

Supplementary Section S15 - Transient junction-temperature simulation under varying duty cycles

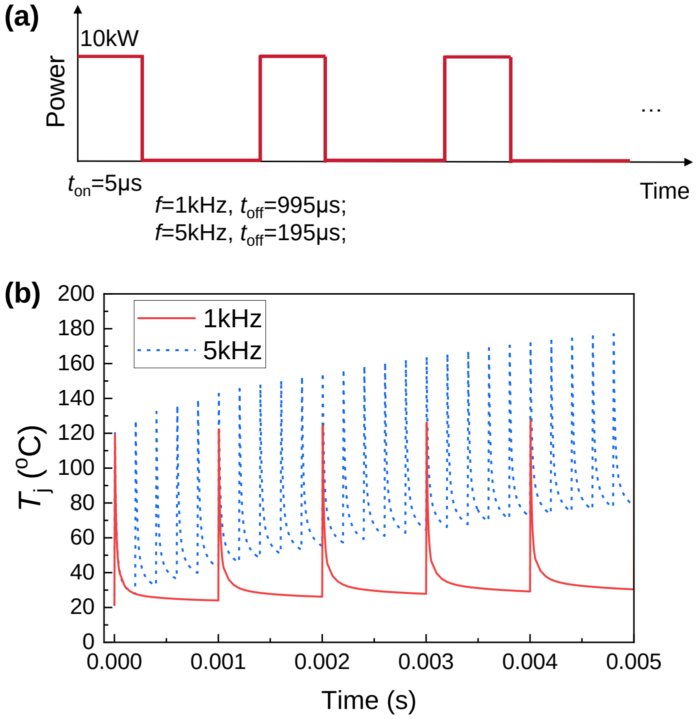

Supplementary Figure S15. Junction temperature simulation in repetitive pulsed-power switching. (a) Simulation setup schematic of applied power dissipation at different repetition rate. (b) Simulated transient junction-temperature responses.

The repetition rate (or duty cycle) directly determines the average power dissipation of the module and therefore its thermal capacity. For a given pulse energy, increasing the repetition rate can increase the average heat load, while at low duty cycles the thermal response is dominated by transient heating rather than steady-state temperature rise. To evaluate the impact of repetition rate (or duty cycle), we performed transient electro-thermal simulations using ANSYS, in which the Ga_2O_3 power sub-module is mounted on a backside heat sink and cooled by forced air (fan cooling).

To explicitly study the repetition-rate effect, the applied power dissipation was modeled as a square-wave pulse, with a peak power of 10 kW and pulse width of 5 μs**.** Two repetition rates were considered: 1 kHz (duty cycle = 1/200) and 5 kHz (duty cycle = 1/40). The simulation setup schematic is shown in Fig. S15a. The corresponding transient junction-temperature (T_j) responses are compared in Fig. S15b. Owing to the large simulation data volume, only the first 5 ms of junction-temperature evolution is presented.

At 1 kHz, the average power dissipation is very small; thus, the average junction temperature barely increases, and the peak transient temperature rise is governed by the thermal capacitance of the device and package. When the repetition rate increases to 5 kHz, heat accumulation becomes pronounced because the generated heat cannot be fully dissipated between pulses. As a result, an elevated junction temperature develops in addition to the repetitive transient temperature peaks. While the transient peaks are still determined by the thermal capacitance of the device and package, the average junction temperature rise is strongly influenced by long-timescale heat removal, including junction-side cooling and the thermal conductivity of the packaging materials in our sub-module structure.

Supplementary Section S16 - Material CTE and power cycling test

Table S3. The list of key material CTE.

Material	CTE (10^{-6}/K)
Sintered Ag	~20
SiO_2	~0.55
$BaTiO_3$	10.8-17.5
Ga_2O_3	3.77 (a), 7.8 (b), 6.34 (c)

Table S3 summarizes the coefficient of thermal expansion (CTE) of Ga_2O_3, SiO_2, $BaTiO_3$, and sintered Ag. Owing to the large CTE mismatch among Ga_2O_3, SiO_2, and sintered Ag, the Ga_2O_3/SiO_2/Ag stack in sub-module A experiences pronounced thermo-mechanical stress at the die edges. In contrast, replacing SiO_2 with $BaTiO_3$ improves the CTE match in the $Ga_2O_3/BaTiO_3$/Ag stack, thereby mitigating edge-induced stress concentration.

To evaluate long-term reliability under realistic thermal-electrical stress conditions, we performed a switching-based power cycling test, which is one of the widest used method for industrial module qualification[18]. Fig. S16a and Fig. S16b show the schematic and photo of switching-based power cycling circuit. Initially, S1 and S3 are turned on, generating a high current within the rating range of the DUT. This current flows through the loop formed by S1, S3, *L*, and the DUT. Next, S1 is turned off and S2 is turned on, forcing the current to freewheel through the loop consisting of S2, L, and the DUT. During this process, the freewheeling time is relatively long, gradually heating the DUT. By repeating this cycle, the DUT can be gradually heated to the target temperature within 60 seconds. The circuit then stops operation, allowing the DUT to cool down to room temperature. This heating-cooling cycle is repeated to achieve power cycling. Fig. S16c presents the injected power profile applied to device and case temperature profile. In the subsequent 120-s cooling period, a forced-air convection is applied to rapidly dissipate accumulated heat. Case temperature evolution is continuously monitored using an infrared thermal-imaging camera, and the peak case temperature reaches ~100 ^{o}C.

At the end of each cooling stage, we capture the current and voltage waveforms under the same excitation conditions and reconstruct a dynamic I-V curve by extracting a series of (I, V) points, each taken at the same time instant (Fig. S16d). From the re-constructed dynamic I-V characteristics, the forward-voltage drop (V_F) is extracted at a fixed current of 5 A. Fig. S16e shows that sub-module C exhibits minimal V_F shift over ~10,000 power-cycling cycles, indicating stable conduction characteristics. In addition, and we did not observe measurable degradation in reverse blocking capability. In contrast, sub-module A exhibits serious V_F degradation after ~1,200 cycles, suggesting the possible formation of delamination, crack, and voids in the package interface. This contrast proves that sub-module C with BTO interface demonstrates the high structural and electrical robustness under dynamic switching stress.

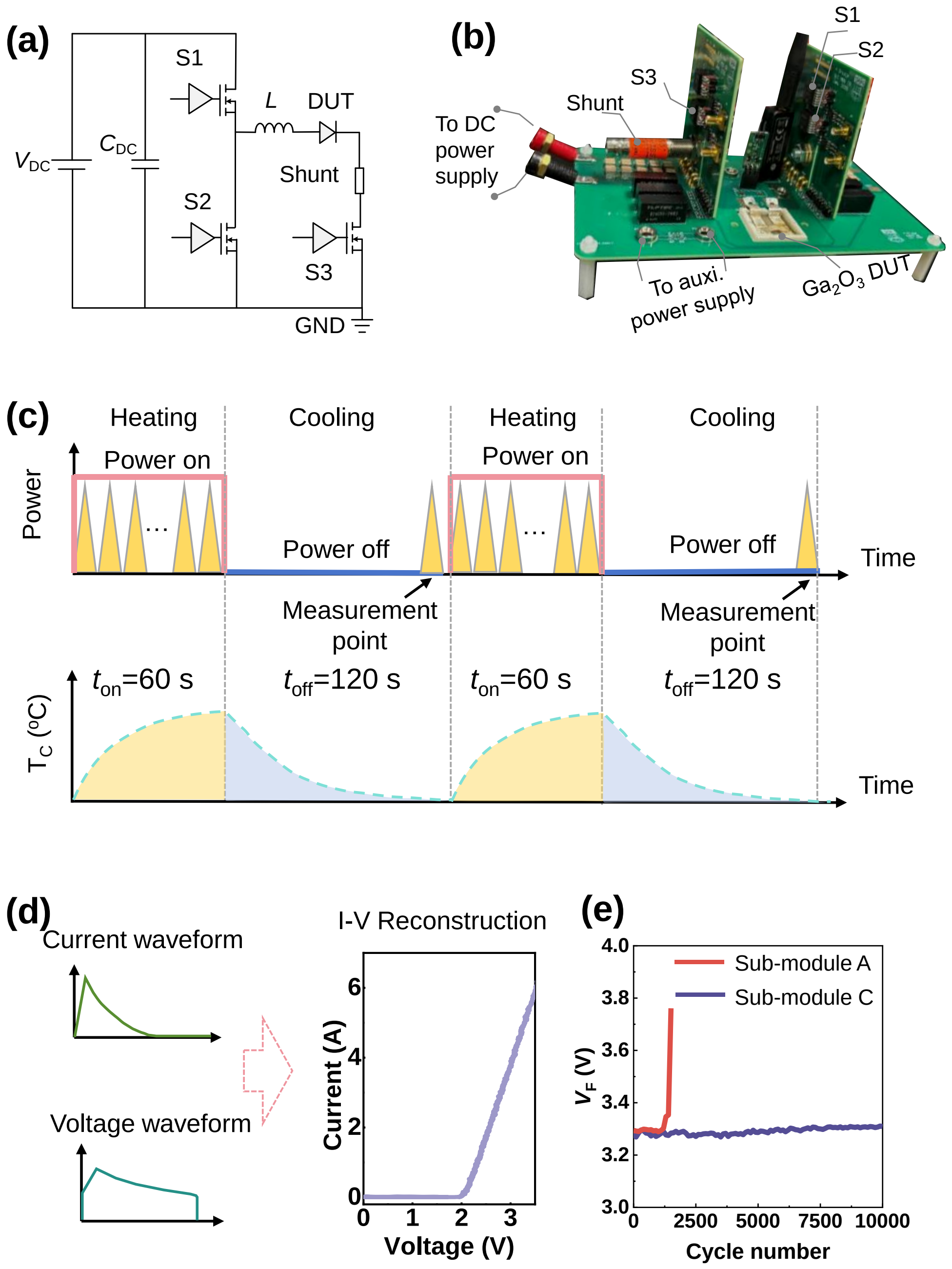

Figure S16. (a) Schematic and (b) photo of power cycling test circuit. (c) Injected power profile applied to device and case temperature profile. (d) Illustration of dynamic I-V reconstruction from the switching current and voltage waveforms recorded in each switching cycle. (e) Extracted V_F during power cycling test for sub-module A with SiO_2 interface and sub-module C with BTO interface.

References

1 Sharma, R. *et al.* Effect of probe geometry during measurement of >100 A Ga_2O_3 vertical rectifiers. *Journal of Vacuum Science & Technology A: Vacuum, Surfaces, and Films* **39**, 013406 (2021).

2 Yang, J., Ren, F., Tadjer, M., Pearton, S. J. & Kuramata, A. Ga_2O_3 Schottky rectifiers with 1 ampere forward current, 650 V reverse breakdown and 26.5 MW.cm^{-2} figure-of-merit. *AIP Advances* **8**, 055026 (2018).

3 Otsuka, F. *et al.* Large-size (1.7 × 1.7 mm^2) β-Ga_2O_3 field-plated trench MOS-type Schottky barrier diodes with 1.2 kV breakdown voltage and 10^9 high on/off current ratio. *Applied Physics Express* **15**, 016501 (2021).

4 Hao, W. *et al.* High-Performance Vertical β-Ga_2O_3 Schottky Barrier Diodes Featuring P-NiO JTE with Adjustable Conductivity. in *2022 International Electron Devices Meeting (IEDM).* 9.5.1-9.5.4 (IEEE, 2022).

5 Guo, W., Han, Z., Zhao, X., Xu, G. & Long, S. Large-area β-Ga_2O_3 Schottky barrier diode and its application in DC–DC converters. *Journal of Semiconductors* **44**, 072805 (2023).

6 Roy, S. *et al.* Ultra-low reverse leakage in large area kilo-volt class β-Ga_2O_3 trench Schottky barrier diode with high-k dielectric RESURF. *Applied Physics Letters* **123**, 243502 (2023).

7 Feng, Y. *et al.* Statistical Study of Large-Area Schottky Barrier Diodes Fabricated on 2-in β-Ga_2O_3 Wafer Using Au-Free Processes. *IEEE Transactions on Electron Devices* **72**, 1528-1532 (2025).

8 Gong, H. *et al.* 70-μm-Body Ga_2O_3 Schottky Barrier Diode With 1.48 K/W Thermal Resistance, 59 A Surge Current and 98.9% Conversion Efficiency. *IEEE Electron Device Letters* **43**, 773-776 (2022).

9 Lv, Y. *et al.* Demonstration of β-Ga_2O_3 Junction Barrier Schottky Diodes With a Baliga's Figure of Merit of 0.85 GW/cm^2 or a 5A/700 V Handling Capabilities. *IEEE Transactions on Power Electronics* **36**, 6179-6182 (2021).

10 Wu, F. *et al.* Superior performance β-Ga_2O_3 junction barrier Schottky diodes implementing p-NiO heterojunction and beveled field plate for hybrid Cockcroft–Walton voltage multiplier. *IEEE Trans. Electron Devices* **70**, 1199-1205 (2023).

11 Wei, J. *et al.* Experimental Study on Electrical Characteristics of Large-Size Vertical β-Ga_2O_3 Junction Barrier Schottky Diodes. in *34th International Symposium on Power Semiconductor Devices and ICs (ISPSD).* 97-100 (IEEE, 2022).

12 Gong, H. *et al.* Enhanced Avalanche (2.1 kV, 83 A) in NiO/Ga_2O_3 Heterojunction by Edge Termination Optimization. *IEEE Electron Device Letters* **45**, 1421-1424 (2024).

13 Gong, H. *et al.* 1.37 kV/12 A NiO/β-Ga2O3 Heterojunction Diode With Nanosecond Reverse Recovery and Rugged Surge-Current Capability. *IEEE Transactions on Power Electronics* **36**, 12213-12217 (2021).

14 Zhou, F. *et al.* 1.95-kV Beveled-Mesa NiO/β-Ga_2O_3 Heterojunction Diode With 98.5% Conversion Efficiency and Over Million-Times Overvoltage Ruggedness. *IEEE Transactions on Power Electronics* **37**, 1223-1227 (2022).

15 Kimoto, T., Yamada, K., Niwa, H. & Suda, J. Promise and Challenges of High-Voltage SiC Bipolar Power Devices. *Energies* **9**, 908 (2016).

16 Schweitzer, D., Pape, H., Chen, L., Kutscherauer, R. & Walder, M. Transient dual interface measurement — A new JEDEC standard for the measurement of the junction-to-case thermal resistance. in *2011 27th Annual IEEE Semiconductor Thermal Measurement and Management Symposium.* 222-229 (IEEE, 2011).

17 Wang, B. *et al.* Low Thermal Resistance (0.5 K/W) Ga_2O_3 Schottky Rectifiers With Double-Side

Packaging. *IEEE Electron Device Letters* **42**, 1132-1135 (2021).
18 Zhang, Y. *et al.* Power Cycling Testing for Power Semiconductor Switches: Methods, Standards, Limitations, and Outlooks. *IEEE Trans. Power Electron.* **41**, 849-869 (2026).
